# Validation of the Chinese version of the exercise empowerment scale among college students (EES-C)

**DOI:** 10.3389/fpsyg.2024.1349310

**Published:** 2024-04-26

**Authors:** Shaojing Weng, Junfeng Yuan, Lin Luo

**Affiliations:** School of Physical Education, Guizhou Normal University, Guiyang, China

**Keywords:** college students, EES, validity, reliability, exercise empowerment

## Abstract

**Objective:**

The escalating prevalence of physical inactivity among college students represents a significant public health challenge, particularly in light of its correlation with detrimental health outcomes. A growing body of evidence underscores the necessity of adhering to recommended levels of regular physical activity to thwart the onset of chronic diseases. One primary aim of school physical education initiatives is to elevate physical activity levels and bolster student motivation toward engaging in physical exercise. Engagement in sports activities has demonstrated efficacy in augmenting students’ motor skills, elevating their self-efficacy, and enhancing cognitive competencies related to physical prowess, while also promoting sustained participation in physical activities. The Exercise Empowerment Scale (EES) has been formulated to quantitatively assess the degree of exercise empowerment. To date, no validated Chinese version of the EES has been reported in the literature. To address this, this present study developed and validated a Chinese version of the EES in a large sample of Chinese college students.

**Methods:**

A sample of Chinese college students (*n* = 885) was analyzed using Rasch analysis to examine the validity of the Exercise Empowerment Scale. In addition, physical activity and psychological resilience were used to investigate the potential associations with exercise empowerment.

**Results:**

The EES-C was found to be a unidimensional scale, and the distribution of item difficulty was relatively even. The scale had good reliability (individual reliability of 0.87, and item reliability of 0.99). No differential item functioning (DIF) was observed across genders for any of the 13 EES-C items, suggesting equitable and unbiased applicability for both male and female respondents. The five-point scoring method of the EES scale was consistent with the overall distribution of the items and subjects. Exercise empowerment was positively correlated with autonomous physical activity and psychological resilience.

**Conclusion:**

The results of the study indicate that the EES-C possesses robust psychometric properties, rendering it suitable for application among Chinese college student populations. The adapted version of the EES-C provides a basis for further exploration of the predictive factors of physical activity in Chinese samples. The generalizability of our findings should be further verified in other populations in the future.

## 1 Introduction

The World Health Organization (WHO) emphatically underscores the multifarious benefits of regular physical activity, accentuating its pivotal role in mitigating hypertension, enhancing glucose sensitivity, and curtailing the incidence of heart disease. These endeavors constitute an integral component of global public health initiatives, aimed to counteracting the burgeoning burden of non-communicable diseases (World Health Organization, 2010). Notwithstanding the well-documented advantages, a pronounced discrepancy persists between the recommended levels of physical activity and actual participation rates, particularly among adolescents and university students. This chasm is disconcerting, as substantial evidence indicates that a majority of these populations fail to meet the minimum activity guidelines, underscoring a pervasive public health challenge (Keating et al., 2005; Brand and Ekkekakis, 2018). Consequently, the WHO has intensified efforts to promote physical activity across various demographics, albeit with mixed success due to significant barriers in maintaining consistent exercise regimens, leading to notable attrition rates from physical activity programs (Razon and Tenenbaum, 2014).

Recent scholarly works have discussed the concept of “empowerment through exercise,” conceptualizing it as a pathway to gain strength, encouragement, and motivation, thereby enhancing adherence to physical activity regimens (Conger and Kanungo, 1988; Blinde et al., 1993; LaVoi, 2007). Empirical evidence substantiates the significant influence of physical education on cultivating exercise habits and its vital role in fostering health awareness and maintaining physical fitness among students. Furthermore, studies have revealed a positive correlation between favorable experiences in physical education classes and increased student participation in physical activities, suggesting that increasing enjoyment and intrinsic motivation through these programs could significantly bolster regular exercise adherence (Ryan and Deci, 2006; Hu et al., 2021). The development of the Exercise Empowerment Scale (EES) by Moore and Fry (2014, 2017) and colleagues represents a pivotal advancement in quantifying the construct of exercise empowerment and elucidating its psychological underpinnings, providing critical insights into mechanisms for enhancing exercise adherence and, consequently, improving public health outcomes.

However, the absence of a culturally adapted Chinese version of the EES highlights a significant gap, especially considering the high prevalence of chronic diseases and suboptimal physical activity levels in China, underscoring an urgent need for tailored research and intervention strategies in this context (Yan, 2010). This study endeavors to bridge this gap by developing and validating a Chinese version of the EES, thereby examining its relationship with exercise behavior and psychological resilience among Chinese college students. It hypothesizes that exercise empowerment positively correlates with both exercise behavior and psychological resilience, suggesting that the EES could play a crucial role in enhancing engagement in physical activity and improving mental health outcomes. This research aims to contribute novel insights into the dynamics of exercise empowerment and its implications for the physical and psychological well-being of the Chinese college student population, thereby adding a significant dimension to the global discourse on health promotion through physical activity.

## 2 Methods

### 2.1 Participants

This study utilized the “Maike” online survey platform to conduct an anonymous questionnaire survey of college students across China. In order to ensure the representativeness of the survey and adhere to ethical standards, we rigorously screened the participants. Qualified participants had to be physically healthy and proficient in Chinese, ensuring their ability to accurately understand and complete the questionnaire.

After meticulous screening, which included the removal of participants with unusually rapid response times or uniform answers, a total of 885 valid responses were retained for analysis. Informed consent was obtained from all participants prior to their involvement in the study. The research protocol, identified as GZNUPEI20220524, received approval from the Ethics Committee of Guizhou Normal University.

### 2.2 Measures

The Empowerment in Exercise Scale (EES) was employed to evaluate empowerment in exercise (Moore and Fry, 2017). This unidimensional scale comprises 13 items, such as “My confidence to do physical activities/exercises on my own has increased.” Ratings on each item are based on a Likert-type scale, ranging from 1 (strongly disagree) to 5 (strongly agree). A higher aggregate score signifies an elevated level of perceived empowerment in exercise. The English version of the scale exhibits commendable internal consistency, as indicated by a Cronbach’s α value of 0.890.

Physical activity levels were assessed using the Physical Activity Rating Scale (PARS3) (Liang, 1994). This scale consists of three sections, comprising a total of 14 items, designed to measure the time individuals spent engaging in physical activities of varying intensities (low, moderate, and high) over the preceding 7 days. Participants were required to record the specific types of physical activities they engaged in (e.g., running) and report the frequency and duration of these activities on a weekly and daily basis. For instance, “How often did you engage in moderate-intensity activities (such as brisk walking, playing sports, etc.) last week?” The calculation of the physical activity level involves multiplying the scores across three dimensions (intensity × time × frequency), with higher scores indicating higher levels of physical activity. The scale exhibited good internal consistency, with a Cronbach’s α coefficient of 0.84 reported in the original author’s research.

Psychological resilience was assessed using the Chinese version of the Connor-Davidson Resilience Scale (CD-RISC). Developed by American psychologists Connor and Davidson (2003) and later adapted for the Chinese context by Yu and Zhang (2007), this scale particularly emphasizes the self-efficacy dimension of resilience. It comprises 8 items, each rated on a scale from 1 (not at all true) to 5 (true nearly all the time). An example item from the scale is ‘I can deal with whatever comes,’ reflecting the individual’s ability to cope with challenges. Higher aggregate scores on this scale indicate stronger psychological resilience. The Chinese adaptation of the CD-RISC has shown robust internal consistency, evidenced by a Cronbach’s α value of 0.910.

### 2.3 Procedures

The testing process followed rigorous scientific translation protocols and involved multiple stages. First, the research team contacted the author of the Exercise Empowerment Scale (EES) and obtained permission to develop a Chinese version. They then assembled a translation team comprising individuals proficient in both Chinese and English. Next, two native Chinese-speaking researchers independently translated the original English version into Chinese. Afterward, they compared and discussed the translations to resolve any discrepancies, resulting in a unified initial Chinese version. Subsequently, two English professors, unaware of the original English version, independently back-translated the initial Chinese version into English. This back-translation ensured a high degree of semantic consistency between the back-translated and original versions.

During the testing process, researchers randomly selected 585 university students from 15 universities across the country as participants. The average age was 21.7 years, with a balanced gender ratio. As the study targeted undergraduate college students, with the overwhelming majority falling within the age range of 18 to 24, the representation of those aged 27 and 28 was inherently limited. Therefore, the random sampling process naturally yielded only a minimal number of participants from these two age groups. Researchers ensured that the participants had a certain level of exercise experience and physical fitness. In the data collection phase, standardized instructions and demonstrations were provided to ensure participants accurately understood the questions. Participants then completed paper-based questionnaires on-site, with staff available to address any queries. Each participant answered independently, and supervision was provided to prevent any interaction among participants. To ensure data quality, three dedicated proctors supervised the questionnaire administration process. After completion, 20% of the questionnaires were randomly inspected for any irregularities or violations. Invalid and unqualified questionnaires were excluded, resulting in 528 valid responses.

### 2.4 Statistical analysis

Data analysis was executed using two software programs, SPSS25.0 and Winsteps, for comprehensive statistical analysis. Within SPSS25.0, several analyses were performed, including: (i) demographic statistics analysis (covering gender, age, place of residence, year level, and major); (ii) normality tests and item discrimination analysis for the Chinese version of the Exercise Empowerment Scale (EES-C); (iii) Kaiser-Meyer-Olkin (KMO) test and Bartlett’s test of sphericity; (iv) exploratory factor analysis (EFA) on the EES-C to evaluate its unidimensionality; and (v) correlation analysis between exercise empowerment, physical activity level, and psychological resilience.

In Winsteps, the analyses encompassed: (i) principal component analysis (PCA) of the Rasch model residuals to assess the unidimensionality of the scale, with criteria for unidimensionality being a standardized residual eigenvalue of the first principal component less than 3.0, at least 40% of the variance explained by the measurement dimension (latent trait), and a ratio of the explained variance of the measurement dimension to the first principal component’s residual variance exceeding 3:1; (ii) model fit testing for the EES-C, with a mean square (MNSQ) value between 0.5–1.5 indicating an appropriate fit; (iii) reliability testing for the EES-C, considering a reliability index of at least 0.7 acceptable and above 0.8 as good; (iv) differential item functioning (DIF) analysis by sex, where an absolute DIF Contrast absolute value greater than 0.5 and a *p*-value less than 0.05 signify; and (v) evaluation of the rating scale of the EES-C, ensuring each option meets the criteria of having at least 10 observations, a unidimensional distribution, monotonic mean category values, an Outfit MNSQ below 2.0, and adjacent category difficulty differences of at least 0.81 logits for the 5-point rating scale. These analyses were pivotal in ensuring the comprehensive assessment of the measurement validity of the EES-C.

## 3 Results

### 3.1 Participant characteristics

Table 1 presents the characteristics of the participants. The survey encompassed college students within the age range of 18–28 years, with a mean age of 19.55 (SD = 1.258). Among the participants, 41.20% identified as male, while 58.80% were female. Regarding ethnicity, 55.80% of the sample belonged to the Han ethnic group, while 44.20% represented various minority groups. A significant majority, 83.80%, originated from rural areas. In terms of academic level, the distribution was as follows: 43.50% first-year students, 50.70% second-year students, 2.70% third-year students, and 3.10% fourth-year students. The academic disciplines of the participants varied, comprising 24.00% humanities students, 39.20% science students, 33.00% engineering students, and 3.80% students from the arts and sports fields.

**Table 1:** Demographic information.

	Variable	M ± SD	Option	Frequency	Percentage (%)
Sample (*n* = 885)	Gender	0.590 ± 0.493	Male	365	41.20%
			Female	520	58.80%
	Age (years)	19.550 ± 1.257	18	183	20.70%
			19	288	32.50%
			20	251	28.40%
			21	103	11.60%
			22	43	4.90%
			23	10	1.10%
			24	5	0.60%
			27	1	0.10%
			28	1	0.10%
	Nationality	0.440 ± 0.497	Han	494	55.80%
			Others	391	44.20%
	Registered residence	0.840 ± 0.368	City	143	16.20%
			Rural	742	83.80%
	Grade	1.650 ± 0.681	Freshman	384	43.40%
			Sophomore	450	50.80%
			Junior	24	2.70%
			Senior	27	3.10%
	Major	2.170 ± 0.834	Humanities and Social Sciences	212	24.00%
			Natural Sciences	348	39.30%
			Engineering	291	32.90%
			Arts and Sports	34	3.80%

M, Mean; SD, Standard Deviation.

### 3.2 Project analysis

The study conducted an analysis of the mean, standard deviation, skewness, kurtosis, and *p*-value for the correlations among the 13 items of the Exercise Empowerment Scale (EES), as detailed in Table 2. The skewness coefficients for the EES items displayed absolute values ranging from 0.436 to 1.452. Since all values were less than 3, this indicates that the skewness of each item was within the critical range, consistent with a normal distribution (Gao et al., 2012). Similarly, the kurtosis coefficients exhibited absolute values between 0.005 and 4.329, all falling below the threshold of 10. This suggests that the kurtosis for each item was within the acceptable range and met the established statistical requirements (Gao et al., 2012). Furthermore, the correlation matrix for all items, as depicted in Table 2, revealed that the correlations between all items were statistically significant.

**Table 2:** EES-C normality test.

Item	Average value	Standard deviation	Skewness	Kurtosis
Q1	3.33	1.008	−0.433	−0.247
Q2	3.44	0.920	−0.624	0.143
Q3	3.49	0.899	−0.815	0.544
Q4	3.47	0.974	−0.720	0.005
Q5	3.56	0.903	−0.915	0.639
Q6	3.65	0.850	−0.905	0.802
Q7	3.61	0.854	−0.654	0.501
Q8	3.61	0.873	−0.908	0.995
Q9	3.88	0.796	−1.357	3.121
Q10	3.94	0.695	−1.179	3.578
Q11	3.95	0.728	−1.452	4.329
Q12	3.93	0.716	−1.329	3.885
Q13	3.64	0.889	−0.875	0.656

In order to analyze the discriminative power of each item, this study implemented the extreme group comparison method. This approach involved dividing the total scores of the 13 items of the Exercise Empowerment Scale (EES) into two groups: a high-scoring group (comprising the upper 25%, totaling 288 participants) and a low-scoring group (comprising the lower 25%, totaling 240 participants). Independent sample *t*-tests were then applied to ascertain the differences between these two groups for each item. Items that demonstrated a *p*-value less than 0.05 and a *t*-value (Critical Ratio, CR) greater than 3 were considered to have significant discriminative ability. The specific outcomes of this analysis are displayed in Table 3. The results indicated that there were significant differences in the scores of each item between the two groups (*p* < 0.001), with *t* (CR value) exceeding 3. This suggests that the items were effective in distinguishing differences in the viewpoints of the subjects, thereby exhibiting good discriminant validity (Wang et al., 2015).

**Table 3:** Distinctiveness of the items.

Item	High subgroup (75%) (*n* = 288)	Low subgroup (75%) (*n* = 288)	*t*-value (CR value)	*p*-value	95% CI	
					Lower limit	Upper limit
Q1	3.99 ± 0.785	2.58 ± 0.956	18.284	<0.001	1.259	1.562
Q2	4.08 ± 0.611	2.62 ± 0.879	21.771	<0.001	1.331	1.595
Q3	4.08 ± 0.570	2.74 ± 0.942	19.269	<0.001	1.202	1.475
Q4	4.15 ± 0.582	2.58 ± 0.943	22.470	<0.001	1.432	1.707
Q5	4.13 ± 0.612	2.71 ± 0.923	20.343	<0.001	1.280	1.554
Q6	4.20 ± 0.502	2.92 ± 0.911	19.518	<0.001	1.155	1.414
Q7	4.20 ± 0.524	2.86 ± 0.880	20.819	<0.001	1.219	1.474
Q8	4.16 ± 0.549	2.85 ± 0.945	19.030	<0.001	1.178	1.45
Q9	4.35 ± 0.528	3.31 ± 0.950	15.155	<0.001	0.906	1.177
Q10	4.32 ± 0.503	3.43 ± 0.874	14.033	<0.001	0.769	1.020
Q11	4.35 ± 0.505	3.43 ± 0.903	14.029	<0.001	0.789	1.047
Q12	4.35 ± 0.506	3.39 ± 0.870	15.144	<0.001	0.838	1.088
Q13	4.13 ± 0.608	2.98 ± 1.000	15.568	<0.001	1.004	1.294

### 3.3 Unidimensionality test

Item Response Theory (IRT) operates on the principle of unidimensionality, an assumption also integral to the Rasch model, a latent trait model based on IRT (Gustafsson, 1980). This assumption posits that a subject’s performance on specific items is primarily influenced by a single latent trait or ability, allowing other influencing factors to be disregarded. Ensuring unidimensionality is a critical prerequisite for conducting Rasch analysis, where the first factor eigenvector must comply with the condition that the second factor eigenvector’s ratio is close to or greater than 3 (Roth et al., 2008).

To validate this assumption, exploratory factor analysis was conducted on the test data of college students using SPSS 25.0 software. The analysis is considered feasible if the Kaiser-Meyer-Olkin (KMO) value is greater than 0.70 and Bartlett’s test of sphericity yields a significance of *p* < 0.01 (Zhang, 2004). The results, as presented in Table 4, indicated a KMO value of 0.909, which is greater than 0.70, and a Bartlett’s sphericity test significance of *p* < 0.001, thereby fulfilling the criteria for exploratory factor analysis.

**Table 4:** KMO and Bartlett’s spherical test results.

KMO Number of sampling tangibles		0.909
Bartlett’s sphericity test	Approximate cardinality	5266.303
	Degree of freedom (df)	78
	Significance	<0.001

Exploratory factor analysis reveals unidimensionality if multiple components are identified, and the initial ratio of the first and second eigenvectors exceeds 3 (Hambleton and Swaminathan, 1985). Table 5 illustrates the total variance explained by the factor with eigenvalues greater than 1, as extracted by principal component analysis. The ratio of the first to the second factor eigenvalues of the EES-C was 3.19, surpassing the threshold of 3, thus meeting the unidimensionality requirements for the ‘unidimensional’ factor structure, making the data appropriate for Rasch model analysis (Roth et al., 2008).

**Table 5:** Explanation of total variance.

Item	Initial Eigenvalue			Extraction of load sum of squares		
	Total	Percentage variance	Cumulative %	Total	Percentage variance	Cumulative %
Q1	5.697	43.824	43.824	5.697	43.824	43.824
Q2	1.787	13.745	57.569	1.787	13.745	57.569
Q3	0.888	6.830	64.398			
Q4	0.763	5.866	70.264			
Q5	0.637	4.898	75.162			
Q6	0.565	4.344	79.506			
Q7	0.497	3.82	83.325			
Q8	0.460	3.538	86.864			
Q9	0.418	3.219	90.083			
Q10	0.363	2.794	92.877			
Q11	0.341	2.626	95.503			
Q12	0.312	2.404	97.906			
Q13	0.272	2.094	100			

To further substantiate the unidimensionality of the EES-C, Principal Component Analysis (PCA) of Rasch model residuals was utilized. According to Linacre (2006), the standardized first-factor residual eigenvalue and the variance explained by measurement are key indicators of data singularity. The standardized first-component residual eigenvalue should be less than 3.0, and the variance explained by the measurement dimension (latent trait) should constitute at least 40% (Linacre, 2006). Moreover, the ratio of the variance explained by the measurement dimension to the variance of the first principal component’s residual should exceed 3:1, elucidating the scale’s unidimensionality (Embretson and Prenovost, 2000). Table 6 shows the variance explained by the measurement dimension of the EES scale at 46.2%, greater than 40.0%, and the standardized first-component residual eigenvalue at 2.9, less than 3.0. The ratio of the variance explained by the measurement dimension to the first principal component’s residual variance is 46.8:12.7, exceeding 3:1. The variance explained by the first principal component’s residual is 12.7%, which is acceptable (Embretson and Prenovost, 2000). Thus, the unidimensionality of the EES-C scale is established, consistent with the findings of Moore and Fry (2017).

**Table 6:** Table of standardised residual variances (in eigenvalues).

	Standardised residual eigenvalues	Explained variance
Total raw variance in observations	24.3	100.00%
Raw variance explained by measures	11.3	46.50%
Unexplned variance in 1st contrast	2.9	12.00%
Unexplned variance in 2nd contrast	1.6	6.70%
Unexplned variance in 3rd contrast	1.2	4.80%
Unexplned variance in 4th contrast	1.2	4.80%
Unexplned variance in 5th contrast	1.0	4.20%

### 3.4 Model fit test

The Rasch model assesses the congruence between observed data and theoretical predictions by evaluating the difference between the theoretical probability of an examinee’s item response and the actual observed data. The key indicators used to verify the model’s fit include measurement, standard error, weighted mean square (infit MNSQ), unweighted mean square (outfit MNSQ), and the point measurement point-measure correlation coefficient.

Wright et al. (1994) have suggested that when the MNSQ value exceeds 1.4 or falls below 0.6, it can be interpreted as indicative of poor item fit. In alignment with Linacre (2006), who proposed that an optimal fit for MNSQ ranges from 0.5 to 1.5, this study adopts these guidelines (Wright et al., 1994; Linacre, 2006). The infit MNSQ values for the 13 items ranged from 0.82 to 1.31, indicating a satisfactory fit with the model. The outfit MNSQ values varied from 0.79 to 1.57, with item 1 marginally exceeding the normative range of 0.50 to 1.50, suggesting a less than ideal fit with the Rasch model predictions. This implies that the actual responses of participants did not align with the model’s projected outcomes, with students at both high and low ability levels potentially answering these questions either correctly or incorrectly. Excluding this item, the remaining 12 items demonstrated a good fit with the model, as detailed in Table 7.

**Table 7:** Parameters of model fit.

Item	Measure (Difficulty estimates)	SE	Infit MNSQ	Outfit MNSQ	Correlation coefficient
Q1	0.74	0.05	1.31	1.57	0.54
Q2	0.52	0.05	0.90	1.02	0.65
Q3	0.46	0.05	0.97	0.96	0.67
Q4	0.41	0.05	0.93	0.94	0.64
Q5	0.27	0.05	0.91	0.96	0.64
Q6	0.16	0.05	0.91	0.87	0.65
Q7	0.14	0.05	0.82	0.79	0.68
Q8	0.09	0.05	1.28	1.30	0.55
Q9	0.04	0.05	0.89	0.86	0.65
Q10	−0.56	0.06	1.09	1.01	0.61
Q11	−0.73	0.06	0.94	0.86	0.61
Q12	−0.77	0.06	0.90	0.86	0.60
Q13	−0.78	0.06	1.09	0.99	0.58

SE, Standard Error.

The standard error in Rasch analysis reflects the stability of an item’s ability level measurement. A lower standard error signifies a more reliable estimation of a student’s ability by the item (Embretson and Prenovost, 2000). As Table 7 illustrates, the error values for the 13 items ranged between 0.05 and 0.06, approximately around 0.10, indicating a small error estimate and stable estimation of student abilities by the items. This conveys that the questionnaire maintains high internal consistency, and the items reliably estimate respondent levels.

The point measurement correlation coefficient measures the extent of alignment between the item and the measurement objective. Higher correlation coefficients indicate closer proximity to the measurement objective. All point measurement correlation coefficients exceeded the acceptable threshold of 0.30, suggesting that the items effectively achieve the measurement objective and have a significant positive correlation with the total scale (Linacre, 2006). The metric values also indicate an even distribution of difficulty across the scale’s items. Thus, these fit indices collectively affirm that the measurement data align well with the Rasch model (Liu et al., 2016), validating the structure of the EES-C developed in this study.

### 3.5 Reliability

The Rasch measurement model mandates the reporting of two distinct reliability indices—individual reliability and item reliability—as well as two specific indices, namely item separation and individual separation. These indices are crucial in reflecting the psychometric properties of a measurement tool (Boone et al., 2014). The item reliability index in the Rasch model evaluates the consistency of item ranking when applied to a similar sample. Simultaneously, the individual reliability index assesses the consistency of individual rankings upon completion of a comparable test. Additionally, the item and individual separation indices provide insight into the reliability of both items and individuals, where a separation coefficient of at least 2.0 is recommended (Boone et al., 2014).

The analysis of the current study revealed that the individual reliability was 0.87, and the individual separation coefficient stood at 2.56. The item reliability reached 0.99, with the item separation coefficient at 9.26. Generally, a reliability index above 0.7 is deemed acceptable, and values above 0.8 are considered good. In this case, both reliability indices closely approach the ideal value of 1, signifying robust internal consistency across all questionnaires (Boone et al., 2014; Bond et al., 2020).

### 3.6 Item function difference test for gender

Measurement invariance is a crucial concept in psychometric evaluation, impacting not only the stability of measurement but also serving as a vital indicator of a scale’s validity (Engelhard, 2013). This principle necessitates that the difficulty level of test questions remains consistent across diverse demographic groups. The invariance of a scale is predominantly assessed via differential item functioning (DIF) analysis, a method focused on the functional disparity in test items. DIF analysis is based on the premise that individuals from different groups, yet possessing similar psychological traits, might have differing probabilities of selecting the same response for a given item (Tu and Dai, 2007). Typically, DIF is identified by comparing the item difficulty across various groups.

In this study, participants were categorized into male and female groups, and the difficulty values for each item on the scale were calculated for these two groups. This approach helps to determine if the items exhibit distinct measurement functions across different demographic groups. There are two widely accepted criteria for DIF analysis (Zwick et al., 1999): (i) an absolute DIF contrast value greater than 0.5, and (ii) an absolute *t*-value greater than 2. Emphasized the importance of the DIF contrast as a primary indicator. Consequently, this study primarily focused on reporting the DIF contrast values. An item is considered to exhibit DIF if the absolute difference in difficulty between genders exceeds 0.5 and the *p*-value is less than 0.05 (Zwick et al., 1999). The presence of DIF in an item implies potential fairness issues, suggesting the need for its possible removal. As indicated in Table 8, none of the 13 items displayed a DIF effect, affirming their equitable applicability to both male and female participants.

**Table 8:** DIF information table.

Item	Gender	
	DIF Contrast (Difficulty Difference)	*p*-value
Q1	0.38	0.0010
Q2	0.27	0.0072
Q3	0.02	0.8403
Q4	0.29	0.0037
Q5	0.02	0.8122
Q6	0.00	1.0000
Q7	0.03	0.7775
Q8	0.00	1.0000
Q9	0.49	0.0000
Q10	0.39	0.0015
Q11	0.36	0.0031
Q12	0.29	0.0188
Q13	0.08	0.4625

### 3.7 Rating scale analysis

Rating scale analysis serves to assess the validity of test items’ rating options, focusing on aspects such as difficulty level, response format, and scoring methodology. For the rating options of each item to be deemed valid, they must adhere to specific criteria (Linacre, 2002): (i) a minimum of 10 observations rating option; (ii) a unidimensional distribution of ratings; (iii) monotonically increasing average measures for each category corresponding with the rating scale levels; (iv) an Outfit Mean Square (Outfit MNSQ) below 2.0; (v) a minimum difficulty difference between adjacent categories of 1.4 logits for a 3-point scale, 1.1 logits for a 4-point scale, and 0.81 logits for a 5-point scale, with a maximum threshold of 5 logits. Should the test results not satisfy these criteria, an adjustment of the rating options is necessary until compliance is achieved.

In this study, the analysis of the ratings for the 13 items, aligned with established rating criteria, confirmed that the 5-point scoring system of the test fulfills the essential requirements. Each option registered a category count exceeding 10. The difficulty values of the test items displayed a progressive increment, ranging from −1.31 to 3.29. The average measures across the 13 items varied from 0.86 to 3.33 logits, with each average exceeding the 0.81 logits threshold; the highest Outfit MNSQ recorded was 1.68, well within the acceptable limit of 2.0. These outcomes are comprehensively detailed in Table 9. The analysis revealed that not only did the number of observations for each option surpass 10, but the Outfit MNSQ also remained below 2.0. Furthermore, the average measures of the options systematically increased with the level of the options, showcasing a relatively uniform increment. Similarly, the difficulty level consistently escalated with the option levels, with the increment being proportionate. These findings suggest that the 5-point scoring system of the EES-C scale harmoniously aligns with the overall distribution of both the items and the subjects.

**Table 9:** Results of the category determination analysis with five-point scoring.

Option results	Frequency of options	Option difficulty values	External fit index	Grade difficulty
Strongly disagree	318 (3%)	−1.31	1.68	NONE
Disagree	919 (8%)	−0.59	1.06	−2.09
Not sure	2,495 (22%)	0.02	0.84	−1.23
Agree	6,475 (56%)	1.17	0.85	−0.34
Strongly agree	1,298 (11%)	3.29	0.99	3.67

### 3.8 Discriminant validity

Table 10 presents positive correlations between exercise empowerment and both physical activity levels and psychological resilience. Furthermore, psychological resilience exhibits a positive correlation with autonomous exercise motivation. These findings provide convergent evidence for the construct validity of the exercise empowerment scale. Specifically, higher levels of physical activity participation are positively associated with higher exercise empowerment, indicating that increased physical activity can enhance individuals’ subjective sense of energy and control over their physical and mental well-being (Moore and Fry, 2014, 2017). On the other hand, higher levels of psychological resilience are positively associated with higher autonomous exercise motivation, reflecting that more resilient individuals are better equipped to overcome barriers to physical activity participation (Vanden Auweele et al., 1997), thereby promoting an overall heightened sense of exercise empowerment.

**Table 10:** Correlation of variables.

	EES-C	Physical activity level	Psychological resilience
EES-C	1		
Physical activity level	0.318**	1	
Psychological resilience	0.442**	0.280**	1

** Correlation is significant at the 0.01 level (2-tailed).

## 4 Discussion

The study systematically validated the Chinese version (EES-C) of the original Exercise Empowerment Scale (EES), aiming to preserve its unidimensional structure and evaluate its validity and reliability in assessing exercise empowerment among Chinese university students. Employing a comprehensive analytical approach, including principal component analysis (PCA) and Rasch model residual exploration, the findings confirmed the EES-C’s normal distribution, effective discriminant validity, and unidimensional structure. Despite a slight deviation in the outfit MNSQ value for one item, it was retained based on contemporary methodological recommendations emphasizing preserving the original item’s intent and the overall scale evaluation goals (Zhang, 2020).

The EES-C demonstrated high internal consistency reliability, with individual and item reliability indices approaching ideal levels. The absence of differential item functioning across genders ensured equitable applicability, and the five-point rating scale was validated for both items and participants. These findings collectively substantiate the EES-C as a robust measure of exercise empowerment within the Chinese university student population.

Further analysis revealed significant positive correlations between exercise empowerment scores and indices of physical activity levels and psychological resilience, supporting Moore and Fry et al.’s conceptualization of exercise empowerment as a multifaceted construct extending beyond immediate exercise benefits to encompass broader well-being implications (Moore and Fry, 2014, 2017). This aligns with existing literature suggesting that childhood physical activity fosters lifelong exercise habits, benefiting cognitive function and overall physical health (Telama et al., 2006; Kjønniksen et al., 2009; Chu et al., 2022). The positive relationship between exercise empowerment and psychological resilience also resonates with research highlighting exercise as a protective factor against stress and adversity (Vanden Auweele et al., 1997; Ning et al., 2021).

In summary, the validation of the EES-C’s effectiveness illuminates the intricate interplay between exercise empowerment, physical activity levels, and psychological resilience, underscoring the importance of promoting exercise empowerment among Chinese university students. Additionally, it provides an opportunity to further investigate the potential mechanisms underlying these relationships.

## 5 Advantages and limitations

This study offers significant contributions to the literature on exercise empowerment, particularly with the development of the EES-C, which addresses a gap in assessment tools for Chinese college students. The large sample size not only enhances the generalizability of the findings but also allows for a more nuanced understanding of the statistical power of the study, providing a solid foundation for the EES-C’s strong internal consistency and model fit. However, the study’s focus on college students, while insightful, limits the broader applicability of the findings. Future research should, therefore, encompass diverse age groups and health conditions to extend the EES-C’s relevance. The omission of variables such as personality traits, exercise preferences, and motivation, and the decision not to re-evaluate the data after excluding item 1 (Q1) due to its suboptimal outfit MNSQ value, represent areas for further exploration to enhance our understanding of exercise empowerment among college students and validate the EES-C’s robustness.

## 6 Conclusion

The findings of this research demonstrate that the EES-C possesses robust psychometric properties, making it a suitable tool for assessing exercise empowerment levels among Chinese college students. The study also reveals a notable association between exercise empowerment and both physical activity levels and psychological resilience. The validity and reliability of the EES-C, as evidenced by these results, confirm its effectiveness as a measurement instrument. This research paves the way for future studies focused on enhancing physical exercise, introducing new avenues for scholarly inquiry.

Nevertheless, the conclusions drawn from this study warrant further exploration and validation in diverse demographic groups, encompassing various ages and health conditions. Such future research endeavors will provide a broader understanding and applicability of the EES-C, extending its relevance beyond the confines of the current study population.

## Data availability statement

The raw data supporting the conclusions of this article will be made available by the authors, without undue reservation.

## Ethics statement

The studies involving humans were approved by this study has been approved by the Ethics Committee of Guizhou Normal University (GZNUPEI20220524). The studies were conducted in accordance with the local legislation and institutional requirements. The participants provided their written informed consent to participate in this study.

## Author contributions

SW: Data curation, Methodology, Software, Validation, Writing – original draft. JY: Investigation, Supervision, Writing – review & editing. LL: Formal analysis, Funding acquisition, Methodology, Resources, Writing – review & editing.

## Appendix

English and Chinese versions of the exercise empowerment scale items.

**Table:**

Item	Item content	项目内容
Q1	My confidence to do physical activities/exercises on my own has increased.	我自己做体育活动/锻炼的信心增强了
Q2	My knowledge of physical activity/exercise has increased.	我对体育活动/锻炼的知识增加了
Q3	I now have a better understanding of the basic concepts (such as timing, movements, vocabulary) and principles for doing physical activity.	我现在对做体育活动的基本概念(如时间、动作、词汇)和原则有了更好的理解
Q4	My confidence in my ability to perform physical activity movements/skills has increased.	我对自己进行身体活动、运动/技能的信心增强了
Q5	My teacher’s feedback helped to increase my confidence to perform physical activity movements/skills.	我老师给我的反馈帮助我增加了进行身体活动运动/技能的信心
Q6	I would now feel comfortable doing physical activity/exercise somewhere else if needed.	如果需要的话，我现在会觉得在其他地方做体育活动很舒服
Q7	I have a better understanding of how to exercise.	我对如何锻炼有了更好的理解
Q8	I have a better understanding of how to be physically active.	我对如何锻炼身体有了更好的理解
Q9	I believe I can have a positive effect on my general physical health (e.g., blood pressure, decreased aches and pains, improved weight).	我相信我可以对我的一般身体健康有积极的影响(例如，血压，减轻疼痛，改善体重)
Q10	I believe I can have a positive effect on my physical appearance (such as increased definition, improved posture, muscle gain, weight loss).	我相信我可以对我的外表有积极的影响(比如增加清晰度，改善姿势，增加肌肉，减轻体重)
Q11	I believe I can have a positive effect on my physical capabilities (such as strength, endurance, agility, flexibility, balance).	我相信我可以对我的身体能力(如力量、耐力、敏捷性、灵活性、平衡性)产生积极的影响
Q12	I believe I can have a positive effect on my mental health (such as attitude, mood, productivity, clear thinking, confidence).	我相信我可以对我的心理健康有积极的影响(比如态度、情绪、生产力、清晰的思维、信心)
Q13	I am not afraid to walk into other fitness facilities/environments to exercise/complete physical activity.	我不怕走进其他的健身设施/环境去锻炼/完成体育活动
